# High stress hyperglycemia ratio predicts adverse clinical outcome in patients with coronary three-vessel disease: a large-scale cohort study

**DOI:** 10.1186/s12933-024-02286-z

**Published:** 2024-06-01

**Authors:** Zheng Qiao, Xiaohui Bian, Chenxi Song, Rui Zhang, Sheng Yuan, Zhangyu Lin, Chenggang Zhu, Qianqian Liu, Wenjun Ma, Kefei Dou

**Affiliations:** 1 State Key Laboratory of Cardiovascular Disease, Beijing, China; 2 Cardiometabolic Medicine Center, National Clinical Research Center for Cardiovascular Diseases, Fuwai Hospital, National Center for Cardiovascular Diseases, Chinese Academy of Medical Sciences and Peking Union Medical College, No. 167A, Beilishi Road, Xicheng District, Beijing, 100037 China; 3 Hypertension Center, Fuwai Hospital, National Center for Cardiovascular Diseases, Chinese Academy of Medical Sciences and Peking Union Medical College, No. 167A, Beilishi Road, Xicheng District, Beijing, 100037 China; 4 National Clinical Research Center for Cardiovascular Diseases, Fuwai Hospital Chinese Academy of Medical Sciences, Shenzhen, Shenzhen, China

**Keywords:** Coronary artery disease, Coronary three-vessel disease, Stress hyperglycemia ratio, Prognosis

## Abstract

**Background:**

Coronary three-vessel disease (CTVD) accounts for one-third of the overall incidence of coronary artery disease, with heightened mortality rates compared to single-vessel lesions, including common trunk lesions. Dysregulated glucose metabolism exacerbates atherosclerosis and increases cardiovascular risk. The stress hyperglycemia ratio (SHR) is proposed as an indicator of glucose metabolism status but its association with cardiovascular outcomes in CTVD patients undergoing percutaneous coronary intervention (PCI) remains unclear.

**Methods:**

10,532 CTVD patients undergoing PCI were consecutively enrolled. SHR was calculated using the formula: admission blood glucose (mmol/L)/[1.59×HbA1c (%)–2.59]. Patients were divided into two groups (SHR Low and SHR High) according to the optimal cutoff value of SHR. Multivariable Cox regression models were used to assess the relationship between SHR and long-term prognosis. The primary endpoint was cardiovascular (CV) events, composing of cardiac death and non-fatal myocardial infarction (MI).

**Results:**

During the median follow-up time of 3 years, a total of 279 cases (2.6%) of CV events were recorded. Multivariable Cox analyses showed that high SHR was associated with a significantly higher risk of CV events [Hazard Ratio (HR) 1.99, 95% Confidence interval (CI) 1.58–2.52, *P* < 0.001). This association remained consistent in patients with (HR 1.50, 95% CI 1.08–2.10, *P* = 0.016) and without diabetes (HR 1.97, 95% CI 1.42–2.72, *P* < 0.001). Additionally, adding SHR to the base model of traditional risk factors led to a significant improvement in the C-index, net reclassification and integrated discrimination.

**Conclusions:**

SHR was a significant predictor for adverse CV outcomes in CTVD patients with or without diabetes, which suggested that it could aid in the risk stratification in this particular population regardless of glucose metabolism status.

**Supplementary Information:**

The online version contains supplementary material available at 10.1186/s12933-024-02286-z.

## Background

Coronary artery disease (CAD) remains a major threat to public health, with coronary three-vessel disease (CTVD) accounting for one-third of the overall incidence of CAD [1, 2]. Furthermore, the mortality rate of patients with CTVD is nearly double that of individuals with single-vessel lesions [3]. The high incidence and mortality rates render CTVD a particularly noteworthy type of CAD in clinical settings.

It is well established that individuals with dysregulated glucose metabolism or diabetes often carry a more severe burden of atherosclerosis and face a higher risk of adverse cardiovascular events [4, 5]. Hyperglycemic over-stress and diabetes lead to ischemic heart disease and adverse cardiovascular outcomes through a series of epigenetic, molecular, and cellular adaptive mechanisms. These mechanisms include microRNA-155 (miR-155) mediated changes in insulin sensitivity [6]; pericoronary fat over-inflammation, which increases the risk of ischemic heart disease in patients with pre-diabetes compared to normoglycemic patients, resulting in poorer outcomes regardless of PCI [7, 8] or thrombus aspiration [9, 10]. Furthermore, hyperglycemic over-stress induces upregulation of Sodium-glucose cotransporter-2 (SGLT2) expression in cardiomyocytes [11] and using SGLT2 inhibitors can improve cardiac function in diabetic cardiomyopathy by reducing JunD expression [12]. Additionally, hyperglycemia-induced over-inflammation and oxidative stress can be seen as the main triggers of atherogenesis and plaque instability with rupture, leading to plaque instability and rupture [13], and adverse cardiovascular outcomes [14]. Notably, these mechanisms could be seen independently from the diabetes status. In summary, hyperglycemic over-stress induces a pro-oxidative/inflammatory status that alters molecular, metabolic, electrical, and mechanical cardiac functions, potentially leading to heart failure with reduced ejection fraction [15].

Moreover, stress hyperglycemia, which was characterized as elevated admission blood glucose (ABG), is associated with mortality in CAD patients [16, 17]. ABG and hemoglobin A1c (HbA1c) are commonly utilized markers for assessing glycemic status. However, HbA1c primarily reflects chronic glycemic levels, while ABG is subject to variations influenced by individual chronic glycemic profiles. Consequently, neither marker adequately captures the condition of stress-induced hyperglycemia. The stress hyperglycemia ratio (SHR), which was calculated by the formula [ABG (mmol/L)]/[1.59×HbA1c (%) − 2.59)] [18, 19], is considered a reflective indicator of state of glucose metabolism and has been reported to be associated with cardiovascular risk and poor prognosis in previous studies [19–21]. However, the association between SHR and the long-term prognosis of CTVD patients undergoing percutaneous coronary intervention (PCI) remains unclear.

In this large-scale retrospective cohort study, we consecutively included 10,532 patients with CTVD who underwent PCI and were subjected to a three-year follow-up. The aim of this study was to evaluate the association between the SHR and the long-term prognosis of patients with CTVD.

## Methods

### Study design and population

This current study was a single-center prospective cohort study. From January 2017 to December 2018, a total of 12,674 patients who underwent PCI for CTVD were consecutively enrolled at Fuwai Hospital, National Center for Cardiovascular Diseases.

**Fig. 1:**
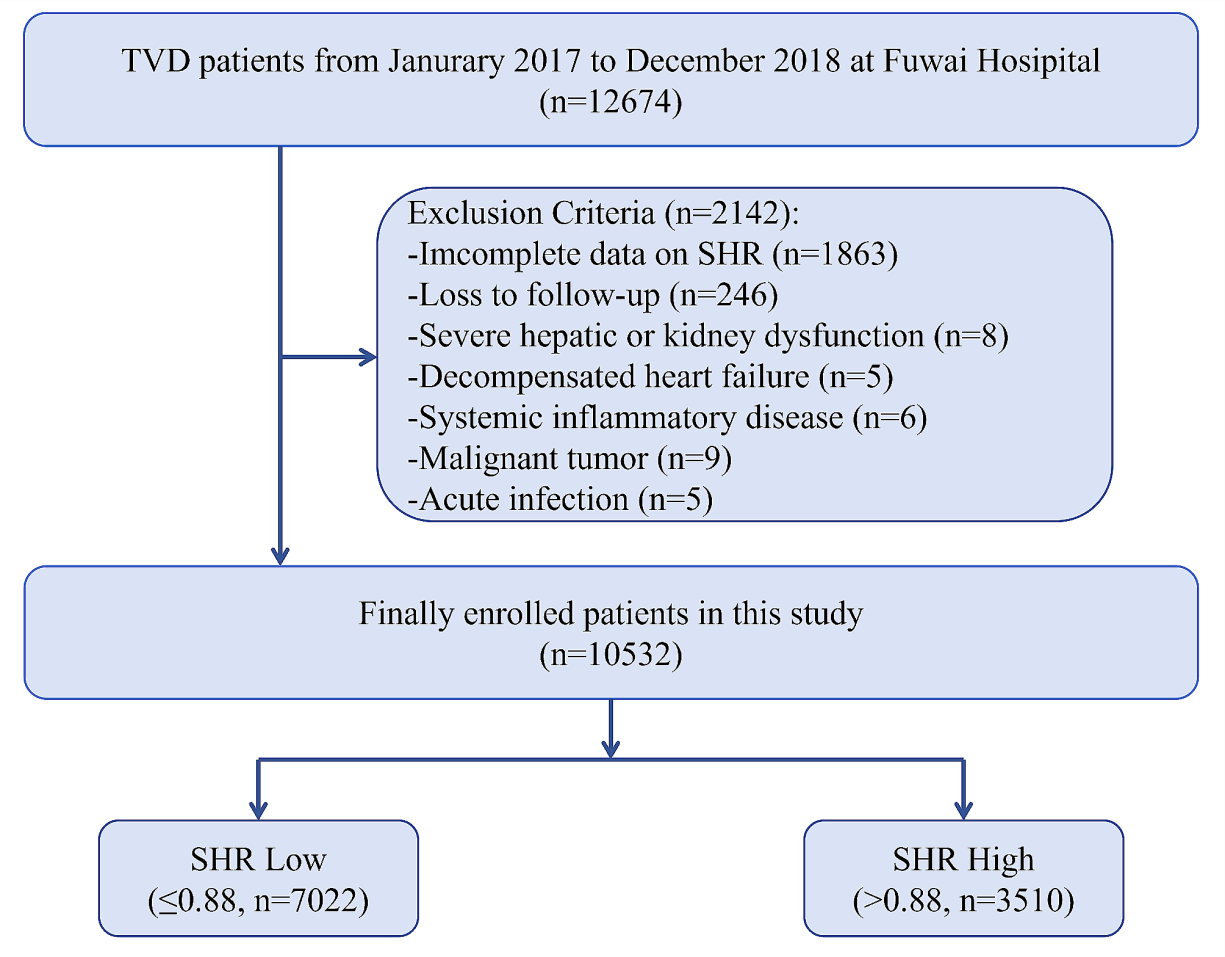
Study flowchart

CTVD was defined as angiographic stenosis ≥ 50% in all 3 main coronary arteries, including the left anterior descending, circumflex, and right coronary artery, with or without involvement of the left main artery [22, 23]. The main exclusion criteria were incomplete data on SHR, severe hepatic or kidney dysfunction, decompensated heart failure, systemic inflammatory disease, malignant tumor, acute infection, and loss to follow-up. Ultimately, a total of 10,532 participants were analyzed in the present study. The detailed flow chart is shown in Fig. 1.

Patients were stratified into two groups based on SHR tertiles: SHR Low (including T1 and T2, ≤ 0.88) and SHR High (including T3, > 0.88). The cutoff value of 0.88 for SHR was consistent with the results obtained from the receiver operating characteristic (ROC) curve analysis. The optimal cutoff values were determined according to the maximum Youden Index, which equals the sum of sensitivity and specificity minus 1.

This study was conducted in compliance with the Declaration of Helsinki and was approved by the Ethics Committee of Fuwai Hospital (2016-847). The informed consent form version number is AS2016-1.1. All participants provided written informed consent for both study participation and interventional procedures before enrollment. Relevant information, including the study’s purpose, procedures, potential risks and benefits, confidentiality measures, and the rights of the participants, was provided to all participants before including them in the study. All participants in this study provided written informed consent before intervention.

### PCI procedure and medication treatment

All PCI procedures and medical therapies were performed in accordance with the recommendations outlined in the guidelines and at the discretion of the cardiologist, as previously detailed [24]. All patients received loading doses of aspirin (300 mg), clopidogrel (600 mg), or ticagrelor (180 mg) before PCI. After the coronary intervention, the characteristics of the coronary disease, including the number of stenotic vessels, unusual types of coronary stenosis, the SYNergy between percutaneous coronary intervention with TAXus and cardiac surgery (SYNTAX) score [25], and data related to stent implantation, were analyzed and recorded by two coronary intervention specialists who were blinded to the baseline data.

### Data collection and definitions

Baseline demographic and clinical characteristics were prospectively collected for all participants in the study. Demographic information encompassed age, sex, body mass index (BMI), concurrent diseases, smoking status, family history of CAD, and previous myocardial infarction (MI) or revascularization history (PCI or coronary artery bypass grafting [CABG]). Clinical data comprised the primary diagnosis upon hospital admission, findings from physical examinations, diagnostic imaging, laboratory analyses, and the pharmacological treatment regimen prescribed at the time of discharge.

Admission blood glucose (ABG) analyses were conducted utilizing LABOSPECT 008 system (Hitachi, Tokyo, Japan), while HbA1c levels were quantified employing high-performance liquid chromatography on a Tosoh G8 HPLC Analyzer (Tosoh Bioscience, Tokyo, Japan). The concentrations of triglycerides (TG), total cholesterol (TC), low-density lipoprotein cholesterol (LDL-C), high-density lipoprotein cholesterol (HDL-C), and creatinine were determined through enzymatic assays using an automated biochemical analyzer (Hitachi 7150, Tokyo, Japan). The measurement of high-sensitivity C-reactive protein (hsCRP) was performed using standard biochemical methodologies at the core laboratory of Fuwai Hospital. Left ventricular ejection fraction (LVEF) at rest was evaluated in accordance with the modified biplane Simpson’s rule [26].

The estimated average chronic glycemic level was calculated using the formula [1.59×HbA1c(%)–2.59] mmol/L [18]. Subsequently, SHR was defined as ABG (mmol/L) divided by the estimated average chronic glycemic value [19].

Diabetes mellitus (DM) status was documented under the following criteria: if the patient had a prior diagnosis of DM, was undergoing glucose-lowering therapy, or exhibited fasting blood glucose levels ≥ 7.0 mmol/L, glycated hemoglobin (HbA1c) levels ≥ 6.5%, or 2-hour plasma glucose levels ≥ 11.1 mmol/L during an oral glucose tolerance test [27, 28]. Hypertension was defined as systolic blood pressure ≥ 140 mmHg, diastolic blood pressure ≥ 90 mmHg, or the use of antihypertensive therapy [29]. Stroke was defined as a previous history of cerebral bleeding, ischemic stroke, or transient ischemic attack; diagnosis of chronic coronary syndrome (CCS) and acute coronary syndrome (ACS) on admission was according to the latest guideline [30, 31].

### Follow-up and end point definitions

Patients were followed-up at 6-month intervals for a duration of 3 years following discharge, utilizing medical records, clinical visits, and/or telephone interviews conducted by trained investigators who were blinded to the patients’ clinical data. The primary endpoint was defined as CV events, a composite of cardiac death and non-fatal MI. The ICD-10 code for cardiac death, specifically for sudden cardiac death, is I46.1. The ICD-10 code for a non-fatal myocardial infarction (heart attack) is I21.4. Secondary endpoint was defined as the two composite events. Death was considered cardiac unless unequivocal non-cardiovascular cause could be established. Nonfatal MI was characterized by the presence of positive cardiac troponins accompanied by typical chest pain, typical serial changes in the electrocardiogram, identification of an intracoronary thrombus through angiography or imaging evidence indicating new loss of viable myocardium or a new regional wall-motion abnormality [32].

### Statistical analysis

Continuous variables were presented as the mean ± standard deviation if they followed a normal distribution; otherwise depicted as the median (interquartile range). Meanwhile, categorical variables were exhibited as frequencies (percentages). Group discrepancies were evaluated utilizing either one-way ANOVA, the Kruskal‒Wallis H test, Pearson’s chi-square test, or Fisher’s exact test, accordingly.

The Kaplan-Meier curves were employed to depict the cumulative incidence of clinical endpoints across different groups, while the log-rank test was utilized to compare these incidences. Hazard ratios (HRs) along with their corresponding 95% confidence intervals (CIs) were calculated using both univariable and multivariable Cox regression models. The multivariable Cox regression model incorporated various potential confounders, including age, male sex, BMI, hypertension, AMI, previous MI, previous PCI, previous CABG, smoking status, previous stroke, LVEF, TC, LDL-C, eGFR, hsCRP, serum creatinine, preprocedural SYNTAX score, calcification, total stent length, aspirin use, clopidogrel use and statins use.

We constructed Restricted Cubic Spline (RCS) plots to examine the linearity assumptions regarding the association between SHR and clinical endpoints. Additionally, in the RCS model, adjustments were made for other confounding factors as detailed above. To evaluate enhancements in risk discrimination, we employed Harrell’s C-statistic, as well as the continuous net reclassification improvement (NRI) and the integrated discrimination improvement (IDI), modified for survival analyses [33, 34]. Statistical significance was defined as two-tailed P values < 0.05. All statistical analyses were conducted using R version 4.0.2 (The R Foundation).

## Results

### Baseline characteristics according to clinical outcomes or SHR levels

Between January 2017 and December 2018, a total of 10,532 CTVD patients were finally included in this study. The average age of the enrolled participants was 60.64 ± 9.89 years old, and 77.6% of them were male. The average value of SHR was 0.86 ± 0.20 in the whole population. Comparisons of the baseline characteristics between participants with and without clinical outcomes were shown in Table 1. Compared with event-free patients, those who suffered from adverse events tended to be older, have higher proportion of comorbidities, including diabetes (higher FBG and HbA1c), heart failure (lower LVEF), renal dysfunction, prior MI and prior CABG, and have higher level of SHR. SHR showed a normal distribution among the total population (Additional file 1: Figure S1). The clinical presentation of patients with events were more likely to be AMI. Regarding the angiographic characteristics, the lesions in patients who developed events during the follow-up period were further complicated by calcification. The baseline characteristics grouped by the levels of SHR were presented in Additional file 1: Table S1. The GLM regression analysis indicated LVEF (*P* < 0.001), Diabetes mellitus (*P* < 0.001), Smoking (*P* = 0.007), Previous MI (*P* = 0.011) were associated with post-PCI QFR value (Additional file 1: Table S2).

**Table 1:** Baseline characteristics according to SHR groups

	Total (*N* = 10,532)	SHR Low (*N* = 7022)	SHR High (*N* = 3510)	*P*-value
Age, years	60.64 ± 9.89	60.66 ± 9.84	60.59 ± 9.99	0.755
Male, n (%)	8172 (77.6)	5404 (77.0)	2768 (78.9)	0.029
BMI, kg/m^2^	26.00 ± 3.16	25.97 ± 3.16	26.06 ± 3.18	0.167
Heart rate per minute	70.83 ± 11.57	70.02 ± 11.24	72.45 ± 12.03	< 0.001
Admission SBP (mmHg)	131.39 ± 17.73	131.25 ± 17.61	131.67 ± 17.95	0.242
Admission DBP (mmHg)	77.36 ± 10.91	77.16 ± 10.86	77.78 ± 11.01	0.006
Smoking, n (%)				0.008
Non smoker	3587 (34.1)	2401 (34.2)	1186 (33.8)	
Former smoker	3641 (34.6)	2362 (33.6)	1279 (36.4)	
Current smoker	3304 (31.4)	2259 (32.2)	1045 (29.8)	
Diabetes, n (%)	4059 (38.5)	2287 (32.6)	1772 (50.5)	< 0.001
Hypertension, n (%)	7136 (67.8)	4694 (66.8)	2442 (69.6)	0.005
Dyslipidemia, n (%)	8303 (78.8)	5536 (78.8)	2767 (78.8)	1
HF, n (%)	248 (2.4)	143 (2.0)	105 (3.0)	0.003
LVEF, %	61.42 ± 7.20	61.78 ± 6.83	60.74 ± 7.79	< 0.001
Prior MI, n (%)	2909 (27.6)	1847 (26.3)	1062 (30.3)	< 0.001
Prior stroke, n (%)	1516 (14.4)	1016 (14.5)	500 (14.2)	0.78
Peripheral vascular disease, n (%)	720 (6.8)	467 (6.7)	253 (7.2)	0.304
Prior PCI, n (%)	2710 (25.7)	1765 (25.1)	945 (26.9)	0.051
Prior CABG, n (%)	391 (3.7)	267 (3.8)	124 (3.5)	0.525
Renal dysfunction, n (%)	250 (2.4)	131 (1.9)	119 (3.4)	< 0.001
Unstable angina, n (%)	4773 (45.3)	3305 (47.1)	1468 (41.8)	< 0.001
AMI, n (%)	1925 (18.3)	1028 (14.6)	897 (25.6)	< 0.001
STEMI, n (%)	1144 (10.9)	581 (8.3)	563 (16.0)	< 0.001
TC, mmol/L	4.06 ± 1.07	4.05 ± 1.05	4.09 ± 1.10	0.15
TG, mmol/L	1.74 ± 1.14	1.70 ± 1.09	1.83 ± 1.24	< 0.001
LDL-C, mmol/L	2.46 ± 0.91	2.45 ± 0.90	2.47 ± 0.94	0.272
HDL-C, mmol/L	1.09 ± 0.28	1.10 ± 0.28	1.08 ± 0.28	0.022
ABG, mmol/L	6.79 ± 2.59	5.83 ± 1.37	8.71 ± 3.29	< 0.001
HbA1C, %	6.60 ± 1.27	6.54 ± 1.20	6.71 ± 1.40	< 0.001
Hemoglobin, g/L	4.75 ± 0.54	4.77 ± 0.52	4.72 ± 0.57	< 0.001
hsCRP, mg/L	2.89 (3.30)	2.81 (3.18)	3.05 (3.52)	0.001
Serum creatinine, umol/L	83.67 ± 20.82	83.32 ± 18.78	84.32 ± 24.23	0.025
**Angiographic and procedural data**				
Pre procedural Syntax	16.22 ± 11.02	15.83 ± 10.44	16.91 ± 11.96	0.005
Calcification, n (%)	4959 (51.0)	3290 (50.7)	1669 (51.5)	0.425
Diffuse lesion, n (%)	6557 (62.3)	4390 (62.5)	2167 (61.7)	0.449
CTO, n (%)	895 (8.5)	621 (8.8)	274 (7.8)	0.078
Pre procedural Minimal lumen diameter, mm	0.36 ± 0.46	0.37 ± 0.44	0.34 ± 0.43	0.002
Lesion length, mm	32.18 ± 20.80	31.99 ± 20.61	32.56 ± 21.18	0.190
Total stent length, mm	35.93 ± 21.07	35.73 ± 20.81	36.31 ± 21.57	0.195
**Medications, %**				
Aspirin	10,174 (96.6)	6799 (96.8)	3375 (96.2)	0.083
Antidiabetic agents	3976 (37.8)	2196 (31.3)	1780 (50.7)	< 0.001
Insulin injection	927(8.8)	519(7.4)	408(11.6)	< 0.001
Oral hypoglycemic agents	3049(28.9)	1677(23.9)	1372(39.0)	< 0.001
Statin	10,204 (96.9)	6794 (96.8)	3410 (97.2)	0.294
Ticagrelor	2169 (20.6)	1356 (19.3)	813 (23.2)	< 0.001
Clopidogrel	8988 (85.3)	6086 (86.7)	2902 (82.7)	< 0.001
CCB	3704 (35.2)	2473 (35.2)	1231 (35.1)	0.899
beta-blockers	9350 (88.8)	6176 (88.0)	3174 (90.4)	< 0.001
**SHR**	0.86 ± 0.20	0.75 ± 0.09	1.07 ± 0.21	< 0.001

Values are mean ± SD or median [25 percentile/75 percentile] or n (%) as accordingly

*PCI* percutaneous coronary intervention, *SYNTAX* Synergy Between Percutaneous Coronary Intervention with Taxus and Cardiac Surgery, *BMI* body mass index, *MI* myocardial infarction, *CAD* coronary artery disease, *PAD* peripheral artery disease, *LVEF* left ventricular ejection faction, *ABG* admission blood glucose, *HbA1c* glycosylated hemoglobin A1c, *TC* total cholesterol, *TG* triglyceride, *HDL-C* high-density lipoprotein cholesterol, *LDL-C* low-density lipoprotein cholesterol, *hsCRP* high-sensitivity C- reactive protein, *SYNTAX* SYNergy between percutaneous coronary intervention with TAXus and cardiac surgery, *CTO* chronic total occlusion, *ACEI* angiotensin converting enzyme inhibitor, *ARB* angiotensin II receptor blocker, *CCB* Calcium channel blockers

### SHR and the long-term prognosis in CTVD patients

**Fig. 2:**
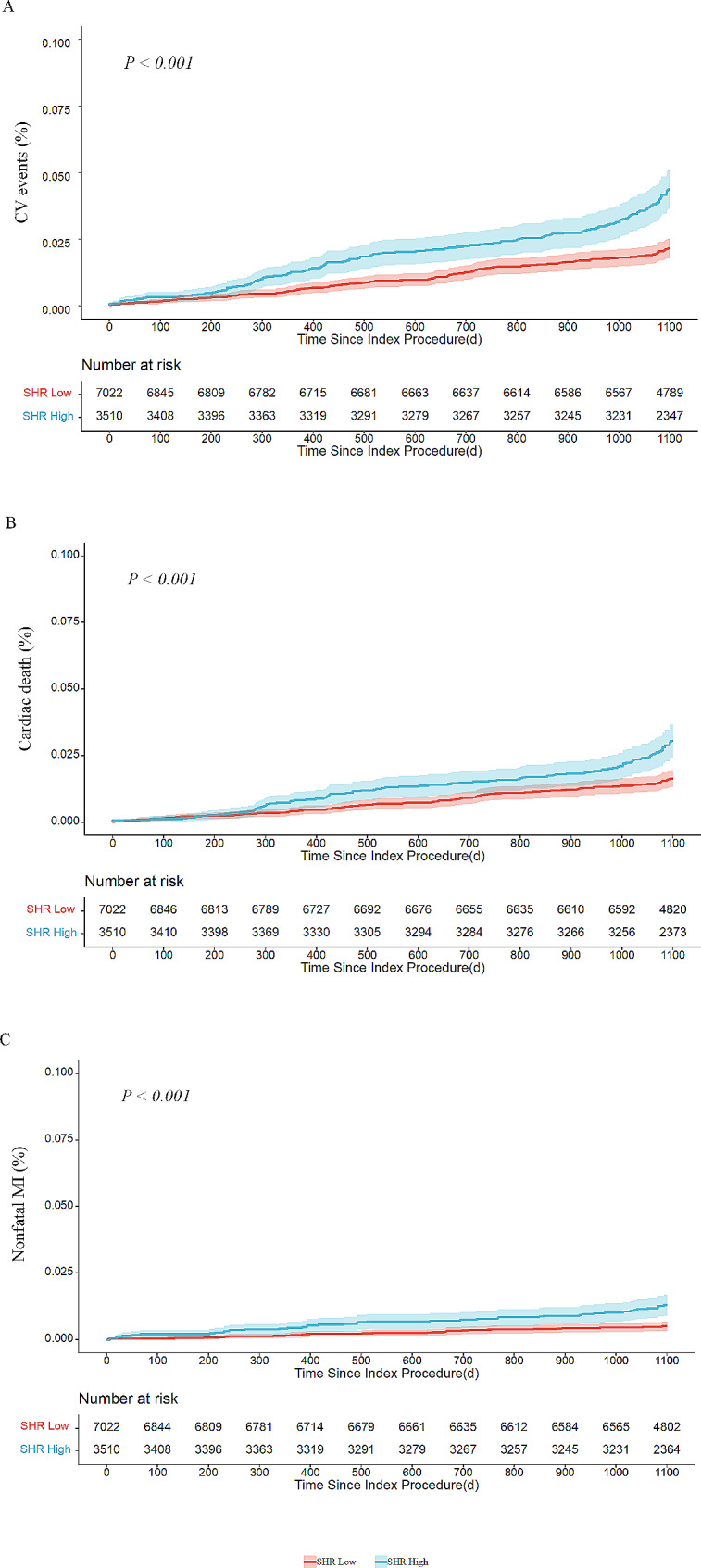
Kaplan–Meier analysis for the cumulative incidence of clinical outcomes according to SHR groups.  **A** CV events **B** Cardiac death **C** Non-fatal MI.  *CV events* cardiovascular events, *SHR* stress hyperglycemia ratio

**Fig. 3:**
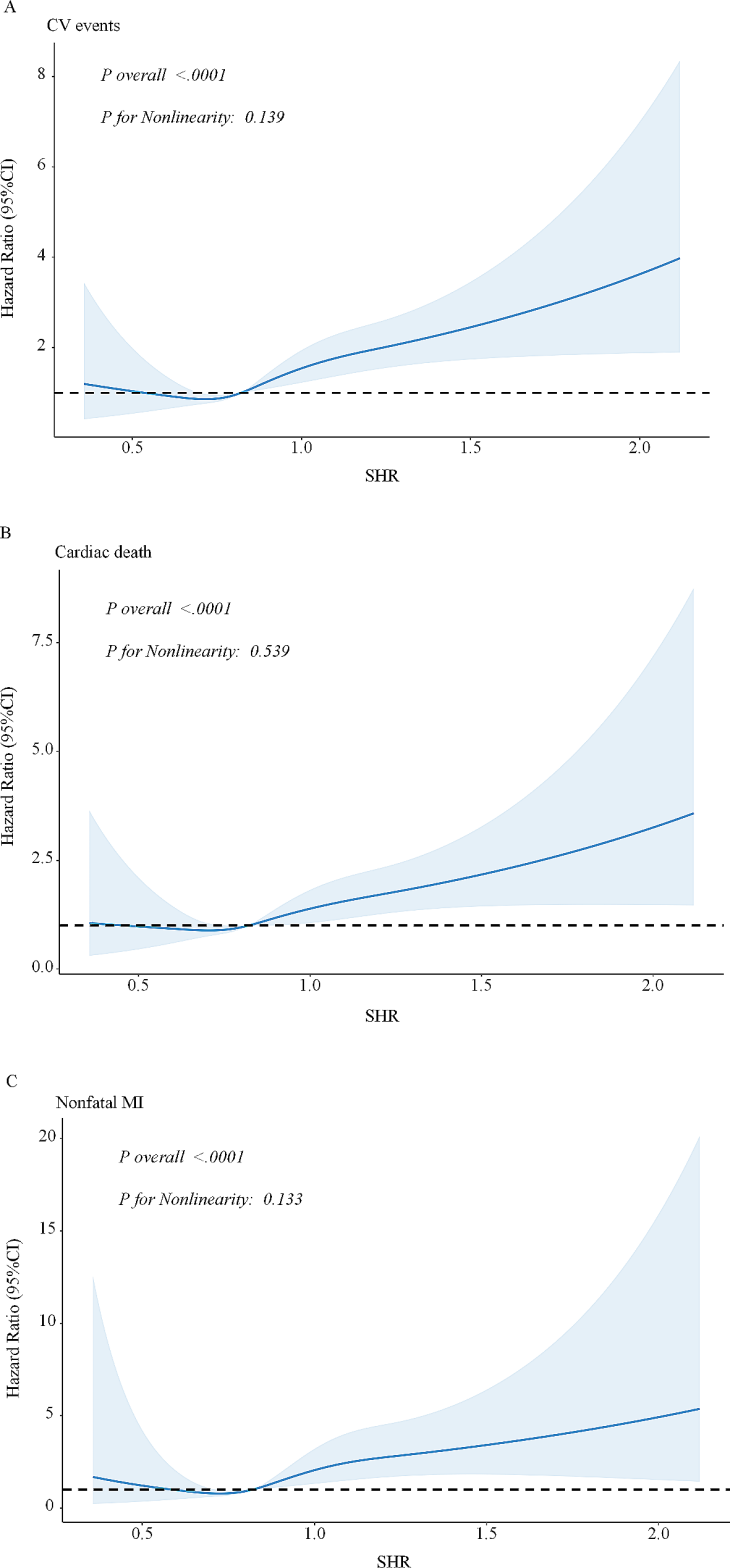
RCS curves for the association of SHR with the risk of clinical outcome.  **A** CV events **B** Cardiac death **C** Non-fatal MI.  *CV events* cardiovascular events, *SHR* stress hyperglycemia ratio, *RSC* restricted cubic spline

During the median follow-up time of 3 years, a total of 279 cases (2.6%) of CV events, including 204 cases (1.9%) of all-cause death and 75 cases (0.7%) of non-fatal MI, were recorded. In the total population, the Kaplan-Meier curves showed a significantly higher incidence of CV events, cardiac death and non-fatal MI in the group of high SHR level (all log-rank *P* < 0.001) (Fig. 2). In the multivariable Cox regression analyses, patients with high levels of SHR had significantly higher risks of CV events (HR 1.99, 95% CI 1.58–2.52, *P* < 0.001), cardiac death (HR 1.81, 95% CI 1.37–2.38, *P* < 0.001) and non-fatal MI (HR 2.26, 95% CI 1.62–4.03, *P* < 0.001) (Table 2). When considered as a continuous variable, SHR (per one-unit increase) was also an independent predictor for CV events (HR 2.53, 95% CI 1.65–3.89, *P* < 0.001), cardiac death (HR 2.15, 95% CI 1.28–3.62, *P* = 0.004) and non-fatal MI (HR 3.66, 95% CI 1.70–7.86, *P* = 0.001) (Additional file 1: Table S3). Restricted cubic spline showed a dose-response relationship between SHR and CV events (*P* for overall < 0.001, *P* for nonlinearity = 0.139), and similar results were observed for cardiac death and non-fatal MI (Fig. 3).

**Table 2:** Association between SHR and clinical outcome

Endpoints	Group		Unadjusted HR (95% CI)	*P* value	Adjusted HR^*^ (95% CI)	*P* value
	SHR Low	SHR High				
Total population	*n* = 7022	*n* = 3510				
CV events	140 (1.9%)	139 (3.9%)	2.01 (1.59, 2.54)	< 0.001	1.99 (1.58, 2.52)	< 0.001
Nonfatal MI	33 (0.4%)	42 (1.1%)	2.57 (1.63, 4.05)	< 0.001	2.26 (1.62, 4.03)	< 0.001
Cardiac death	107 (1.5%)	97 (2.7%)	1.83 (1.30, 2.41)	< 0.001	1.81 (1.37, 2.38)	< 0.001
DM	*n* = 2287	*n* = 1772				
CV events	52 (2.2%)	76 (4.2%)	1.53 (1.10, 2.13)	0.012	1.50 (1.08, 2.10)	0.016
Nonfatal MI	15 (0.6%)	25 (1.4%)	2.16 (1.14, 4.09)	0.019	2.12 (1.12, 4.03)	0.021
Cardiac death	37 (1.6%)	51 (2.8%)	1.78 (1.17, 2.72)	0.007	1.73(1.13, 2.64)	0.011
Non-DM	*n* = 4735	*n* = 1738				
CV events	88 (1.8%)	63 (3.6%)	1.98 (1.47, 2.74)	< 0.001	1.97 (1.42, 2.72)	< 0.001
Nonfatal MI	18 (0.3%)	17 (0.9%)	2.61 (1.35, 5.07)	0.004	2.66 (1.37, 5.12)	0.001
Cardiac death	70 (1.4%)	46 (2.6%)	1.82 (1.25, 2.64)	0.002	1.79 (1.23, 2.59)	0.003

*Adjusted for age, male sex, BMI, hypertension, AMI, previous MI, previous PCI, previous CABG, smoking status, previous stroke, LVEF, TC, LDL-C, hsCRP, serum creatinine, preprocedural SYNTAX score, calcification, total stent length, aspirin use, clopidogrel use and statins use

For DM patients, multivariable Cox analyses revealed a 1.50-fold increased risk for CV events (HR 1.50, 95% CI 1.08–2.10, *P* = 0.016), a 1.73-fold increased risk for cardiac death (HR 1.73, 95% CI 1.13–2.64, *P* = 0.011), and a 2.26-fold increased risk for non-fatal MI (HR 2.12, 95% CI 1.12–4.03, *P* = 0.021) (Table 2; Additional file 1: Figure S2A-C). Per one-unit increase in SHR led to a 2.34-fold increased risk for CV events (HR 2.34, 95% CI 1.37–3.99, *P* = 0.002), a 2.01-fold increased risk for cardiac death (HR 2.01, 95% CI 1.04–3.89, *P* = 0.038), and a 3.13-fold increased risk for non-fatal MI (HR 3.13, 95% CI 1.25–7.87, *P* = 0.015) (Additional file 1: Table S3). There were also observed linear relationships between SHR and the occurrence of CV events, cardiac death, non-fatal MI in the DM population (all P for nonlinearity > 0.05) (Additional file 1: Figure S2A-C). Similar results were noted in patients without DM, except that the continuous SHR was not an independent predictor for cardiac death (HR 2.30, 95% CI 0.99–5.38, *P* = 0.053) (Additional file 1: Table S3).

**Fig. 4:**
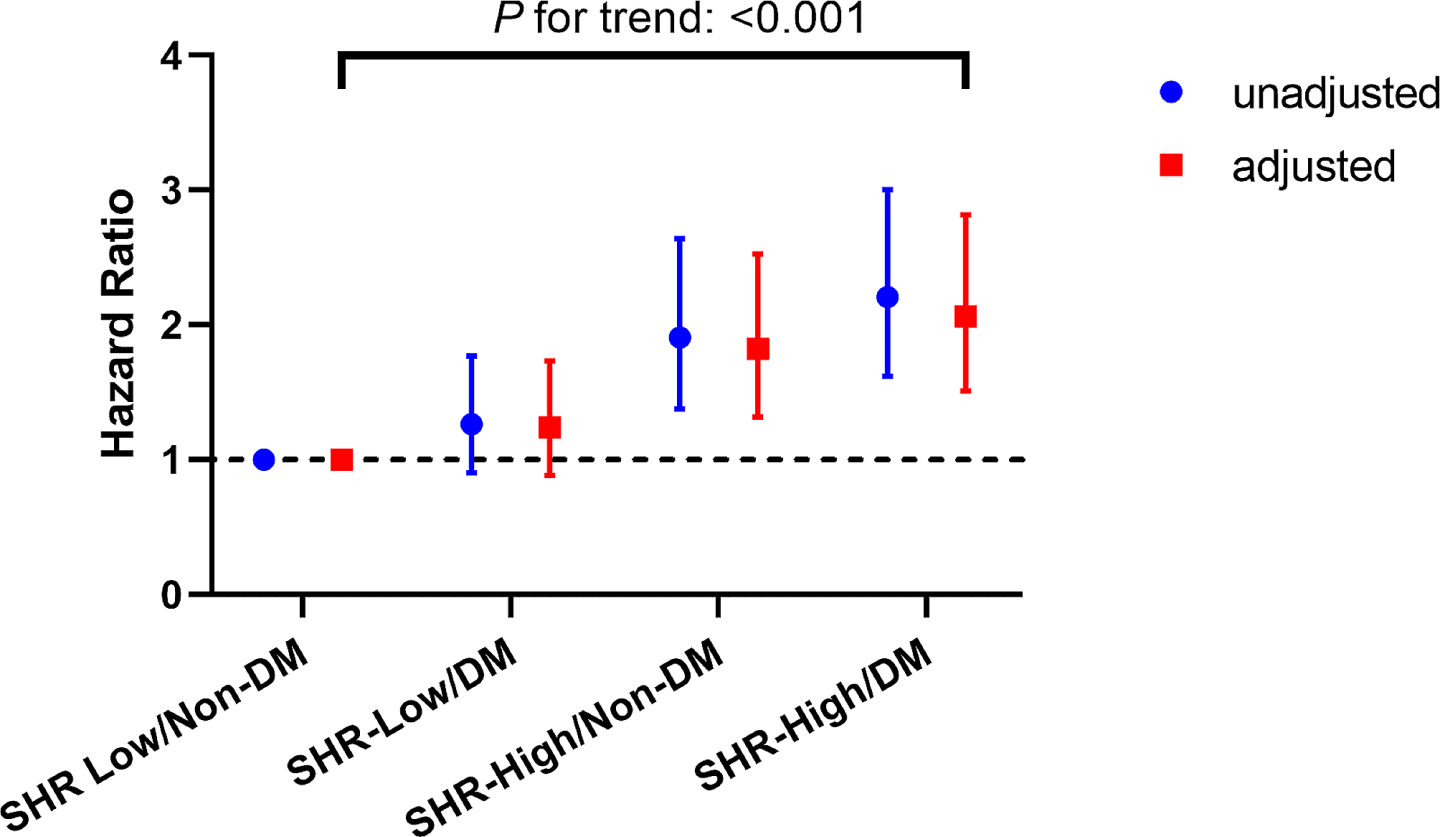
Hazard rations (95% CI) for CV events according to SHR groups and DM status.  *CV events* cardiovascular events, *SHR* stress hyperglycemia ratio, *DM* Diabetes mellitus. Adjusted for age, male sex, BMI, hypertension, AMI, previous MI, previous PCI, previous CABG, smoking status, previous stroke, LVEF, TC, LDL-C, hsCRP, serum creatinine, preprocedural SYNTAX score, calcification, total stent length, aspirin use, clopidogrel use and statins use

After adjusting for potential confounders, DM was an independent predictor for CV events in the total population (HR 1.36, 95% CI 1.08–1.73, *P* = 0.015) (Additional file 1: Table S4). When combined the status of DM and the levels of SHR, the total population were divided into four groups: SHR Low with and without DM, and SHR High with and without DM. As shown in Fig. 4, compared to those in the SHR Low/non-DM group, patients in the high SHR/non-DM and high SHR/DM groups had significantly higher risks of CV events (HR 1.85, 95% CI 1.32–2.53, *P* < 0.001; HR 2.06, 95% CI 1.51–2.82, *P* < 0.001). Among the four groups, patients with high levels of SHR and DM had the highest risk of CV events (*P* for trend < 0.001).

### Predictive value of SHR for CV events in CTVD patients

Introducing SHR into the model of traditional risk factors led to a significant improvement on the predictive ability for CV events. Table 3 indicated that the addition of SHR raised the C-index from 0.676 (95% CI 0.652, 0.691) to 0.694 (95% CI 0.672, 0.701). After adding SHR into the model of traditional risk factors, the NRI (0.83, *P* = 0.038) and IDI (0.18, *P* = 0.008) of CV events were also significantly increased. Similar results were observed in in the DM and non-DM population (Table 3). However, the NRI (0.87, *P* = 0.098) was not significantly increased after the addition of SHR to the base model in DM population.

**Table 3:** C-statistics, NRI, and IDI of SHR for predicting CV events in coronary 3-vessel disease patients

Models	C-statistics (95% CI)	Δ-C-statistics	*P* value	NRI (95% CI)	*P* value	IDI (95% CI)	*P* value
Total population							
Model1	0.676 (0.652, 0.691)	–	Reference	Reference	–	Reference	–
Model1 + SHR	0.694 (0.672, 0.701)	0.018	< 0.001	0.83(0.20,0.92)	0.038	0.18(0.021,0.54)	0.008
DM							
Model1	0.674 (0.642, 0.694)	–	Reference	Reference	–	Reference	–
Model1 + SHR	0.687 (0.655, 0.709)	0.013	0.001	0.87(-0.081,0.96)	0.098	0.41(0.053,0.67)	0.006
Non-DM							
Model1	0.691 (0.672, 0.713)	–	Reference	Reference	–	Reference	–
Model1 + SHR	0.707 (0.672, 0.721)	0.016	0.006	0.17(0.059,0.30)	0.004	0.005(0.001,0.012)	< 0.001

*Adjusted for age, male sex, BMI, hypertension, AMI, previous MI, previous PCI, previous CABG, smoking status, previous stroke, LVEF, TC, LDL-C, serum creatinine, preprocedural SYNTAX score, calcification, total stent length, aspirin use, clopidogrel use and statins use

### Subgroup analysis

Subgroup analyses were conducted to examine the association between SHR and CV events based on age (> 60 years old), sex, hypertension, hyperlipidemia, smoking status, and MI history. The main results remained robust in all subgroups, with no significant interaction observed (Additional file 1: Table S5).

### Sensitivity analysis

To verify the reliability of the relationship between SHR and long-term adverse cardiovascular outcomes in patients with three-vessel disease, we conducted a sensitivity analysis. Based on the median SHR, all 10,532 included patients were divided into two groups: the SHR Below Median group and the SHR Above Median group, with each group comprising 5,266 patients.

In the multivariable Cox regression analyses, patients from SHR Above Median group had significantly higher risks of CV events (HR 1.61, 95% CI 1.27–2.05, *P* < 0.001), cardiac death (HR 1.78, 95% CI 1.11–2.85, *P* = 0.017) and non-fatal MI (HR 1.55, 95% CI 1.17–2.05, *P* = 0.002) (Table S6).

## Discussion

The present study was to investigate the association between SHR and long-term prognosis in patients with or without diabetes who had undergone PCI. For the first time, we found that high SHR was an independent predictor for long time adverse cardiovascular outcomes in CTVD patients, regardless of the diabetes status. When combining the diabetes status and SHR levels, patients with high levels of SHR and DM had the highest risk of CV events. After adding SHR to the base model of traditional risk factors, the C-index, NRI and IDI of the base model significantly improved.

Stress-induced hyperglycemia refers to a state of transient hyperglycemia triggered by the activation of the hypothalamic-pituitary-adrenal axis and the excessive secretion of cortisol and adrenaline in response to stress [35]. Moderate hyperglycemia is a protective mechanism to provide sufficient energy during stressful situations [36]. However, immoderate stress hyperglycemia can have negative effect, and several mechanisms may participate in this pathological process. First, stress-induced hyperglycemia is frequently associated with an upregulation of the immune-inflammatory response, leading to an increase in the release of pro-inflammatory cytokines such as interleukin-1, interleukin-6, and tumor necrosis factor-α [37–40]. Second, hyperglycemia significantly contributes to increased thrombotic activity, enhancing platelet aggregation and fibrinogen levels, thus promoting intra-coronary thrombus formation. This exacerbates adverse outcomes in STEMI patients, including larger infarct sizes and impaired microvascular function [10, 41, 42]. Sigirci et al. showed that in STEMI patients without diabetes undergoing primary PCI, admission hyperglycemia was an independent predictor of large thrombus burden (TIMI grades 4 or 5) [43].

Due to the influence of background glycemic status, admission glucose level could not reflect the extent of stress hyperglycemia exactly. Thus, SHR, calculated by both admission blood glucose and estimated chronic blood glucose, was proposed by Robert et al. [19]. to represent relative hyperglycemia in patients at risk of critical illness. Accumulating studies have demonstrated that SHR was an independent risk factor of CV events in patients with different kinds of cardiovascular diseases, including AMI [44, 45], acute decompensated heart failure [20], severe aortic stenosis [46], ischemia and nonobstructive coronary arteries (INOCA) [47, 48], and acute ischemia or hemorrhagic stroke [49, 50].

Compared to single-vessel disease, CTVD increased the complexity of PCI procedure, and was associated with a worse prognosis [51, 52]. However, the prognostic value of SHR in patients with CTVD has not been investigated yet. For the first time, we found that an increased SHR was associated with elevated CV risks in patients with CTVD, whether they had T2DM or not. In recent years, whether SHR has the same prognostic value in diabetes and non-diabetes still remains a controversial topic. Similar to our results, Cui et al. and Fu et al. found that SHR was an independent predictor for in-hospital mortality and long-term prognosis in AMI patients regardless of diabetes status [53–55]. Interestingly, Zhang et al. found that SHR was positively correlated with the risk of multi-vessel CAD in patients with pre-DM or DM, while no significant correlation was observed between SHR and the incidence of multi-vessel CAD [44]. However, it is worth noting that Zhang’s study only investigated the relationship between SHR and the severity of CAD in a cross-sectional design, without presenting any follow-up data.

The linear or non-linear correlation between SHR and unfavorable prognosis has been argued in the previous studies. Yang et al. demonstrated that there was a U-shaped association between SHR and 2-year adverse prognosis in 5,562 ACS patients [56]. Similarly, in a study of 5,190 ACS patients, a U-shaped association was found between SHR and cardiovascular mortality at 4-year follow-up [21]. As for short-term outcomes, Wei et al. found a J-shaped association between SHR and in-hospital mortality in 1099 patients with STEMI [57]. Conversely, a dose-response relationship was observed between SHR and in-hospital mortality (nonlinear *P* value = 0.260) in a substantial population of 19,929 CAD patients [58]. In the present study, we found a positive linear association between SHR and CV events in patients with CTVD (nonlinear *P* value = 0.139). The inconsistency of this trend in such studies may be due to differences in sample size and population selection across studies. Therefore, further studies are still required to investigate the association between SHR and CV risk in CTVD patients through large-scale, prospective cohort studies or randomized controlled trials (RCTs).

There are several limitations of this study that need to be acknowledged. First, due to the inherent limitation of the cohort study design, undetected confounders may still exist, although all the potential confounders have been adjusted in the multivariable Cox analysis. Second, we initially categorized anti-diabetic medications into insulin injections and oral anti-diabetic drugs. This categorization limited our ability to provide detailed information on the specific types of oral anti-diabetic medications, including incretin treatment like GLP-1 receptor agonists and SGLT2 inhibitors. GLP-1 receptor agonists have been widely used and proven to improve cardiovascular clinical outcome in patients with acute myocardial infarction (NSTEMI and STEMI) treated with PCI [59, 60]. SGLT2 inhibitors are also recognized for improving clinical outcomes through their pleiotropic effects on atherosclerosis of coronary plaque [61, 62]. Third, detailed medication follow-up data were not available in this study. Last but not least, although a relative large-scale population was included, the present study was conducted in a single center of east Asia, the selection bias could not be avoided.

## Conclusions

SHR was a significant predictor for adverse CV outcomes in CTVD patients with or without diabetes, which suggested that it could aid in the risk stratification in this particular population regardless of glucose metabolism status.

### Electronic Supplementary Material

Below is the link to the electronic supplementary material.

Additional file 1

## Data Availability

The datasets used during the current study are available from the corresponding author on reasonable request.

## Abbreviations

BMI: Body mass index
CV: Cardiovascular
CIs: Confidence intervals
DM: Diabetes mellitus
eGFR: Estimated glomerular filtration rate
FBG: Fasting blood glucose
HF: Heart failure
HDL-C: High-density lipoprotein cholesterol
HbA1c: Glycosylated hemoglobin A1c
HRs: Hazard ratios
hs-CRP: High-sensitivity C-reactive protein
LVEF: Left ventricular ejection faction
LDL-C: Low-density lipoprotein cholesterol
PCI: Percutaneous coronary intervention
PAD: Peripheral artery disease
RCSs: Restricted cubic splines
SHR: Stress hyperglycaemia ratio
CTVD: Coronary three-vessel disease
