# Supplementary material for: High stress hyperglycemia ratio predicts adverse clinical outcome in patients with coronary three-vessel disease: a large-scale cohort study
Source: Cardiovasc Diabetol. 2024 Jun 1;23:190. doi: 10.1186/s12933-024-02286-z (PMC11144339; doi:10.1186/s12933-024-02286-z)
Supplement: Supplementary file 1 — Additional file 1 [file 12933_2024_2286_MOESM1_ESM.docx]

**Supplemental materials**

| **Table of Contents** | | **Page Number** |
| --- | --- | --- |
| Table S1 | Baseline Characteristics according to SHR groups. | 2-3 |
| Table S2 | Predictive Value of Characteristics on SHR Value | 4 |
| Table S3 | Prognostic value of continuous SHR value for risk of CV events and other endpoints | 5 |
| Table S4 | Association Between DM and Clinical Outcome | 6 |
| Table S5 | Subgroup analysis for CV events | 7 |
| Table S6 | Association Between SHR and Clinical Outcome according to SHR median | 8 |
| Figure S1 | Distribution of SHR | 9 |
| Figure S2 | Kaplan–Meier analysis for the cumulative incidence of clinical outcomes according to SHR groups in DM and Non-DM patients | 10 |
| Figure S3 | RCS curves for the association of SHR with the risk of clinical outcome in DM and Non-DM patients | 11 |

**Table S1. Baseline Characteristics according to** **clinical outcome.**

|  | **Total**  **(N=10532)** | **Survivors**  **(N=10253)** | **Events**  **(N=279)** | ***P*-value** |
| --- | --- | --- | --- | --- |
| Age, years | 60.64±9.89 | 60.47±9.81 | 66.61±10.94 | <0.001 |
| Male, n (%) | 8172 (77.6) | 7961 (77.6) | 211 (75.6) | 0.468 |
| BMI, kg/m2 | 26.00±3.16 | 26.02±3.15 | 25.08±3.42 | <0.001 |
| Heart rate per minute | 70.83±11.57 | 70.76±11.52 | 73.39±12.85 | <0.001 |
| Admission SBP (mmHg) | 131.39±17.73 | 131.34±17.64 | 133.16±20.52 | 0.09 |
| Admission DBP (mmHg) | 77.36±10.91 | 77.41±10.89 | 75.52±11.80)\ | 0.004 |
| Smoking, n (%) |  |  |  | 0.257 |
| Non smoker | 3587 (34.1) | 3487 (34.0) | 100 (35.8) |  |
| Former smoker | 3641 (34.6) | 3537 (34.5) | 104 (37.3) |  |
| Current smoker | 3304 (31.4) | 3229 (31.5) | 75 (26.9) |  |
| Diabetes, n (%) | 4059 (38.5) | 3931 (38.3) | 128 (45.9) | 0.013 |
| Hypertension, n (%) | 7136 (67.8) | 6942 (67.7) | 194 (69.5) | 0.562 |
| Dyslipidemia, n (%) | 8303 (78.8) | 8090 (78.9) | 213 (76.3) | 0.338 |
| HF, n (%) | 248 (2.4) | 222 (2.2) | 26 (9.3) | <0.001 |
| LVEF, % | 61.42±7.20 | 61.56±7.03 | 56.78±10.55 | <0.001 |
| Prior MI, n (%) | 2909 (27.6) | 2777 (27.1) | 132 (47.3) | <0.001 |
| Prior stroke, n (%) | 1516 (14.4) | 1465 (14.3) | 51 (18.3) | 0.074 |
| Peripheral vascular disease, n (%) | 720 (6.8) | 696 (6.8) | 24 (8.6) | 0.287 |
| Prior PCI, n (%) | 2710 (25.7) | 2624 (25.6) | 86 (30.8) | 0.057 |
| Prior CABG, n (%) | 391 (3.7) | 371 (3.6) | 20 (7.2) | 0.003 |
| Renal dysfunction, n (%) | 250 (2.4) | 223 (2.2) | 27 (9.7) | <0.001 |
| Unstable angina, n (%) | 4773 (45.3) | 4672 (45.6) | 101 (36.2) | 0.002 |
| AMI, n (%) | 1925 (18.3) | 1828 (17.8) | 97 (34.8) | <0.001 |
| STEMI, n (%) | 1144 (10.9) | 1091 (10.6) | 53 (19.0) | <0.001 |
| TC, mmol/L | 4.06±1.07 | 4.07±1.07 | 3.93±1.01 | 0.068 |
| TG, mmol/L | 1.74±1.14 | 1.74±1.15 | 1.57±0.75 | 0.026 |
| LDL-C, mmol/L | 2.46±0.91 | 2.46±0.91 | 2.37 (0.87) | 0.132 |
| HDL-C, mmol/L | 1.09 (0.28) | 1.09 (0.28) | 1.10 (0.28) | 0.71 |
| FBG, mmol/L | 6.79 (2.59) | 6.76 (2.56) | 7.82 (3.47) | <0.001 |
| HbA1C, % | 6.60 (1.27) | 6.59 (1.27) | 6.90 (1.47) | <0.001 |
| Hemoglobin, g/L | 4.75 (0.54) | 4.76 (0.54) | 4.53 (0.67) | <0.001 |
| hsCRP, mg/L | 2.89 (3.30) | 2.88 (3.29) | 3.29 (3.56) | 0.065 |
| Serum creatinine, umol/L | 83.67±20.82 | 83.37±20.33 | 94.10±32.23 | <0.001 |
| **Angiographic and procedural data** |  |  |  |  |
| Pre procedural Syntax | 16.22±11.02 | 16.20±11.03 | 16.84±10.66 | 0.584 |
| Calcification, n (%) | 4959 (51.0) | 4803 (50.7) | 156 (59.3) | 0.007 |
| Diffuse lesion, n (%) | 6557 (62.3) | 6374 (62.2) | 183 (65.6) | 0.271 |
| CTO, n (%) | 895 (8.5) | 876 (8.5) | 19 (6.8) | 0.36 |
| Pre procedural Minimal lumen  diameter, mm | 0.36±0.46 | 0.36±0.47 | 0.32±0.30 | 0.228 |
| Lesion length, mm | 32.18±20.80 | 32.16±20.81 | 32.86±20.46 | 0.582 |
| Total stent length, mm | 35.93±21.07 | 35.91±21.08 | 36.43±20.68 | 0.691 |
| **Medications, %** |  |  |  |  |
| Aspirin | 10174 (96.6) | 9905 (96.6) | 269 (96.4) | 0.996 |
| Antidiabetic agents | 3976 (37.8) | 3841 (37.5) | 135 (48.4) | <0.001 |
| Insulin injection | 927(8.8) | 895(8.7) | 32(11.4) | 0.016 |
| Oral hypoglycemic agents | 3049(28.9) | 2961(28.9) | 88(31.5) | 0.345 |
| Statin | 10204 (96.9) | 9938 (96.9) | 266 (95.3) | 0.183 |
| Ticagrelor | 2169 (20.6) | 2110 (20.6) | 59 (21.1) | 0.876 |
| Clopidogrel | 8988 (85.3) | 8754 (85.4) | 234 (83.9) | 0.537 |
| CCB | 3704 (35.2) | 3611 (35.2) | 93 (33.3) | 0.557 |
| β-blockers | 9350 (88.8) | 9092 (88.7) | 258 (92.5) | 0.059 |
| **SHR** | 0.86±0.20 | 0.84±0.16 | 0.89±0.26 | <0.001 |

Values are mean ± SD or median [25 percentile/75 percentile] or n (%) as accordingly.

PCI, percutaneous coronary intervention; SYNTAX, Synergy Between Percutaneous Coronary Intervention with Taxus and Cardiac Surgery.

*CV* cardiovascular, *BMI* body mass index, *MI* myocardial infarction, *CAD* coronary artery disease, *PAD* peripheral artery disease, *LVEF* left ventricular ejection faction, *ABG* admission blood glucose, *HbA1c* glycosylated hemoglobin A1c, *TC* total cholesterol, *TG* triglyceride, *HDL-C* high-density lipoprotein cholesterol, *LDL-C* low-density lipoprotein cholesterol, *hsCRP* high-sensitivity C- reactive protein,*SYNTAX* SYNergy between percutaneous coronary intervention with TAXus and cardiac surgery, *CCB* Calcium channel blockers

**Table S2. Predictive Value of Characteristics on SHR Value**

|  | **β** | **95% CI** | **P** |
| --- | --- | --- | --- |
| Male | 0.012 | -0.004 to 0.029 | 0.149 |
| Age | 0.000 | -0.001 to 0.001 | 0.664 |
| BMI | 0.000 | -0.001 to 0.001 | 0.504 |
| Diabetes mellitus | 0.056 | 0.001 to 0.044 | <0.001 |
| Hypertension | 0.012 | 0.001 to 0.025 | 0.052 |
| Dyslipidemia | -0.011 | -0.024 to 0.002 | 0.105 |
| Peripheral vascular disease | -0.007 | -0.030 to 0.016 | 0.535 |
| Renal dysfunction | 0.087 | 0.001 to 0.051 | 0.124 |
| Smoking | -0.009 | 0.267 to -0.026 | 0.007 |
| Previous myocardial infarction | 0.017 | 0.004 to 0.030 | 0.011 |
| Previous heart failure | -0.019 | -0.057 to 0.018 | 0.318 |
| Left ventricular ejection fraction | -0.002 | 0.001 to 0.004 | <0.001 |

*Abbreviations as in table S1.*

**Table S3. Prognostic value of continuous SHR value for risk of CV events and other endpoints**

| **Endpoints** | **Unadjusted HR**  **(95% CI)** | **P value** | **Adjusted HR**^*^  **(95% CI)** | **P value** |
| --- | --- | --- | --- | --- |
|  |  |  |  |  |
| Total population |  |  |  |  |
| CV events | 3.38 (2.22, 5.13) | <0.001 | 2.53 (1.65, 3.89) | <0.001 |
| Nonfatal MI | 4.79 (2.35, 9.77) | <0.001 | 3.66 (1.70, 7.86) | 0.001 |
| Cardiac death | 2.88 (1.72, 4.83) | <0.001 | 2.15 (1.28, 3.62) | 0.004 |
| DM |  |  |  |  |
| CV events | 2.85 (1.67, 4.83) | <0.001 | 2.34 (1.37, 3.99) | 0.002 |
| Nonfatal MI | 3.42 (1.41, 8.34) | 0.007 | 3.13 (1.25, 7.87) | 0.015 |
| Cardiac death | 2.60 (1.35, 5.00) | 0.004 | 2.01(1.04, 3.89) | 0.038 |
| Non-DM |  |  |  |  |
| CV events | 3.81 (1.81, 8.03) | <0.001 | 2.89 (1.40, 5.87) | 0.004 |
| Nonfatal MI | 6.30 (1.68, 23.63) | 0.006 | 5.52 (1.36,22.39) | 0.017 |
| Cardiac death | 3.15 (1.29, 7.71) | 0.012 | 2.30 (0.99, 5.38) | 0.053 |

*Adjusted for age, male sex, BMI, hypertension, AMI, previous MI, previous PCI, previous CABG, smoking status, previous stroke, LVEF, TC, LDL-C, hsCRP, serum creatinine, preprocedural SYNTAX score, calcification, total stent length, aspirin use, clopidogrel use and statins use.

**Table S4. Association Between DM and Clinical Outcome**

| **Endpoints** | **Group** | | **Unadjusted HR**  **(95% CI)** | **P value** | **Adjusted HR**^*^  **(95% CI)** | **P value** |
| --- | --- | --- | --- | --- | --- | --- |
|  | **Non-DM**  **(n=6473)** | **DM**  **(n=4059)** |  |  |  |  |
| CV events | 151(2.3%) | 128(3.1%) | 1.35 (1.07, 1.71) | 0.012 | 1.36 (1.08, 1.73) | 0.015 |
| Nonfatal MI | 35(0.5%) | 40(0.9%) | 1.82 (1.16, 2.87) | 0.010 | 1.62 (1.01, 2.58) | 0.044 |
| Cardiac death | 116(1.7%) | 88(2.1%) | 1.21 (0.91, 1.59) | 0.186 | 1.14 (0.86, 1.52) | 0.369 |

*Adjusted for age, male sex, BMI, hypertension, AMI, previous MI, previous PCI, previous CABG, smoking status, previous stroke, LVEF, TC, LDL-C, hsCRP, serum creatinine, preprocedural SYNTAX score, calcification, total stent length, aspirin use, clopidogrel use and statins use.

**Table S5 Subgroup analysis for CV events**

| **Variables** | **Unadjusted Model** | | **Adjusted Model*** | | ***P* for interaction** |
| --- | --- | --- | --- | --- | --- |
|  | **SHR Low** | **SHR High** | **SHR Low** | **SHR High** |  |
| **Age** |  |  |  |  | 0.882 |
| <65 | 1.00 (reference) | 1.93(1.21-3.07) | 1.00 (reference) | 2.36 (0.91-6.12) |  |
| ≥65 | 1.00 (reference) | 2.06 (1.57-2.70) | 1.00 (reference) | 2.72 (1.69-4.37) |  |
| **Sex** |  |  |  |  | 0.696 |
| Male | 1.00 (reference) | 2.04 (1.56-2.67) | 1.00 (reference) | 2.71 (1.65-4.45) |  |
| Female | 1.00 (reference) | 1.93 (1.20-3.11) | 1.00 (reference) | 2.87 (1.25-6.60) |  |
| **Current Smoker** |  |  |  |  | 0.763 |
| No | 1.00 (reference) | 1.96 (1.33-2.88) | 1.00 (reference) | 1.81 (1.21-2.70) |  |
| Yse | 1.00 (reference) | 2.03 (1.51-2.73) | 1.00 (reference) | 2.29 (1.44-3.65) |  |
| **Hypertension** |  |  |  |  | 0.831 |
| No | 1.00 (reference) | 1.96 (1.28-2.30) | 1.00 (reference) | 2.49 (1.44-3.95) |  |
| Yes | 1.00 (reference) | 2.03 (1.53-2.69) | 1.00 (reference) | 4.57 (1.99-10.49) |  |
| **Dyslipidemia** |  |  |  |  | 0.152 |
| No | 1.00 (reference) | 2.80 (1.72-4.60) | 1.00 (reference) | 2.79 (1.70-4.57) |  |
| Yes | 1.00 (reference) | 1.82 (1.39-2.38) | 1.00 (reference) | 2.56 (1.57-4.16) |  |
| **Prior MI** |  |  |  |  | 0.786 |
| No | 1.00 (reference) | 1.84 (1.33-2.54) | 1.00 (reference) | 1.74 (1.25-2.42) |  |
| Yes | 1.00 (reference) | 2.05 (1.46-2.89) | 1.00 (reference) | 1.94 (1.37-2.74) |  |

*Adjusted for age, male sex, BMI, hypertension, AMI, previous MI, previous PCI, previous CABG, smoking status, previous stroke, LVEF, TC, LDL-C, hsCRP, serum creatinine, preprocedural SYNTAX score, calcification, total stent length, aspirin use, clopidogrel use and statins use.

**TableS6. Association Between SHR and Clinical Outcome according to SHR median**

| **Endpoints** | **Group2** | | **Unadjusted HR**  **(95% CI)** | **P value** | **Adjusted HR**^*^  **(95% CI)** | **P value** |
| --- | --- | --- | --- | --- | --- | --- |
|  | **SHR Below Median** | **SHR Above Median** |  |  |  |  |
| Total population | **n=5266** | **n=5266** |  |  |  |  |
| CV events | 109(2.1%) | 170(3.2%) | 1.57 (1.24, 2.00) | <0.001 | 1.61 (1.27, 2.05) | <0.001 |
| Nonfatal MI | 27(0.5%) | 48(0.9%) | 1.79 (1.12, 2.87) | 0.015 | 1.78 (1.11, 2.85) | 0.017 |
| Cardiac death | 82(1.6%) | 122(2.3%) | 1.50 (1.13, 2.41) | 0.005 | 1.55 (1.17, 2.05) | 0.002 |
| DM | **n=1747** | **n=2312** |  |  |  |  |
| CV events | 40(2.3%) | 88(3.8%) | 1.68 (1.16, 2.44) | 0.007 | 1.66 (1.14, 2.42) | 0.008 |
| Nonfatal MI | 12(0.7%) | 28(1.2%) | 1.78 (0.91, 3.50) | 0.095 | 1.76 (0.90, 3.47) | 0.100 |
| Cardiac death | 28(1.6%) | 60(2.6%) | 1.63 (1.04, 2.56) | 0.032 | 1.62(1.03, 2.54) | 0.036 |
| Non-DM | **n=3519** | **n=2954** |  |  |  |  |
| CV events | 69(1.9%) | 82(2.8%) | 1.42 (1.04, 1.97) | 0.030 | 1.48 (1.08, 2.04) | 0.016 |
| Nonfatal MI | 15(0.4%) | 20(0.7%) | 1.60 (0.82, 3.13) | 0.168 | 1.61 (0.82, 3.15) | 0.164 |
| Cardiac death | 54(1.5%) | 62(2.1%) | 1.38 (0.96, 1.98) | 0.086 | 1.45 (1.01, 2.09) | 0.047 |

*Adjusted for age, male sex, BMI, hypertension, AMI, previous MI, previous PCI, previous CABG, smoking status, previous stroke, LVEF, TC, LDL-C, hsCRP, serum creatinine, preprocedural SYNTAX score, calcification, total stent length, aspirin use, clopidogrel use and statins use.

**Figure S1. Distribution of SHR**

**
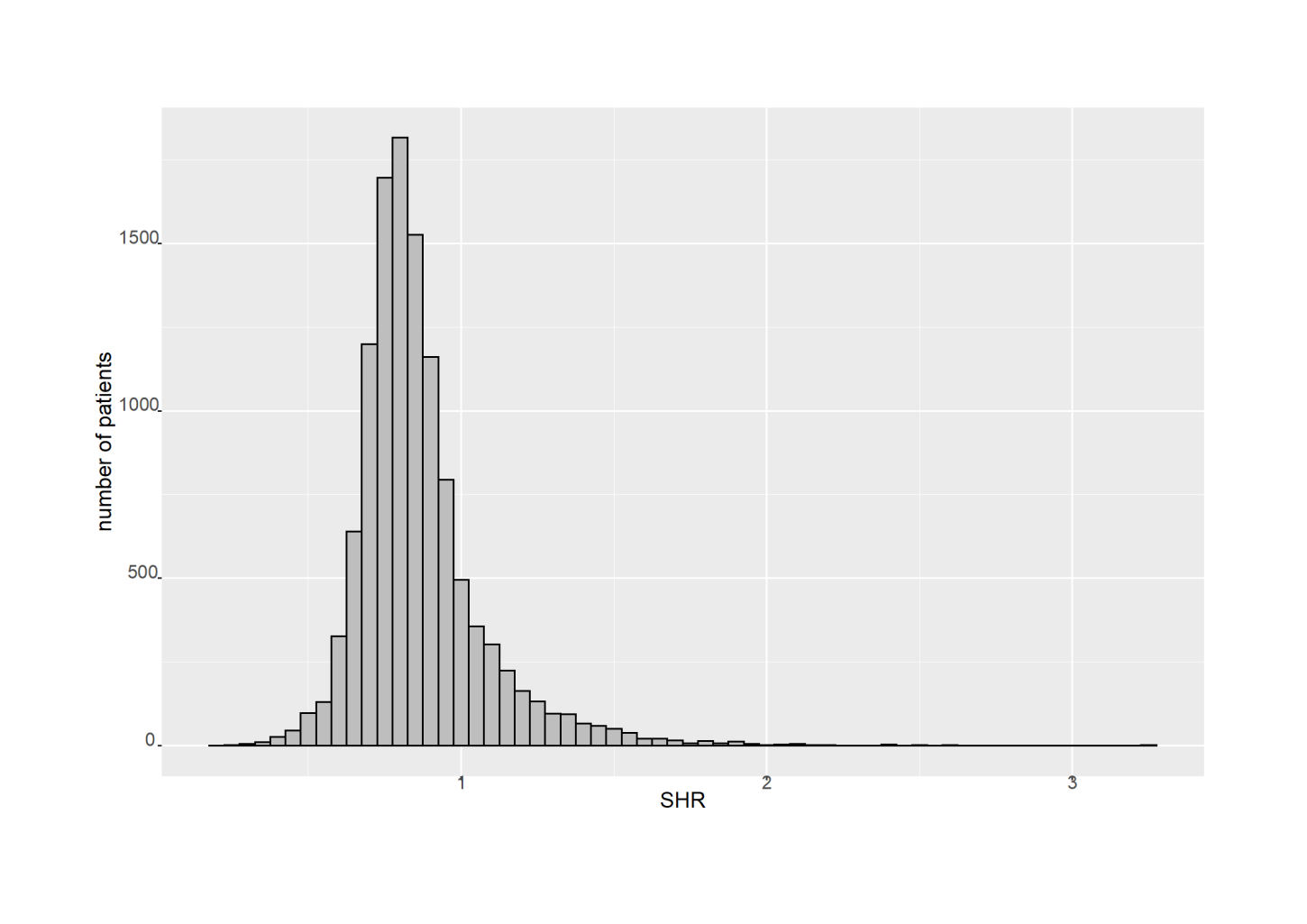
**

SHR stress hyperglycemia ratio

**Figure S2. Kaplan–Meier analysis for the cumulative incidence of clinical outcomes according to SHR groups in DM and Non-DM patients**

**
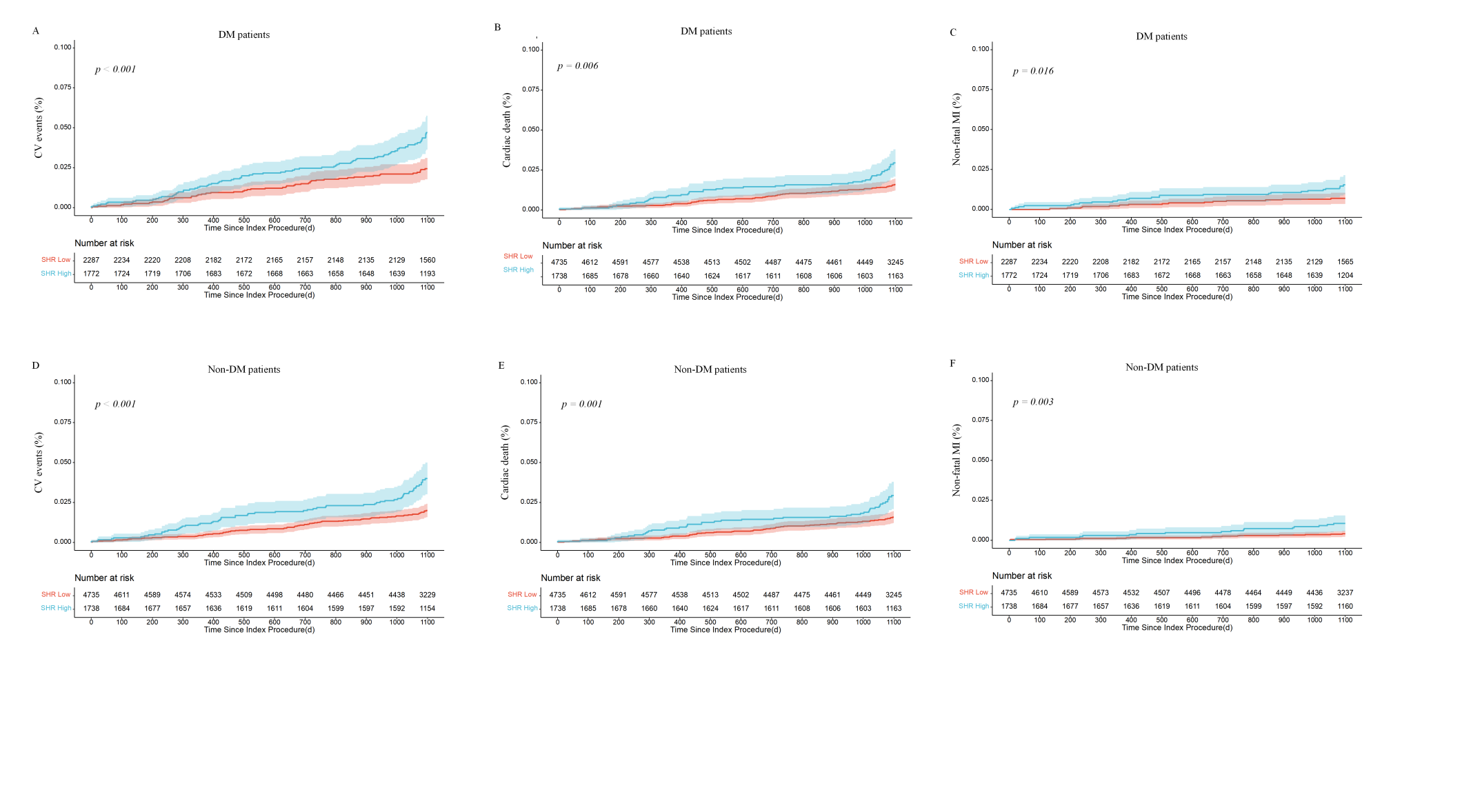
**

Kaplan–Meier analysis for the cumulative incidence of (A), CV events (B), Cardiac death (C). Non-fatal MI in DM patients; (D), CV events (E), Cardiac death (F). Non-fatal MI in Non-DM patients; CV events cardiovascular events, SHR stress hyperglycemia ratio, DM diabetes mellitus

**Figure S3. RCS curves for the association of SHR with the risk of clinical outcome in DM and Non-DM patients**

**
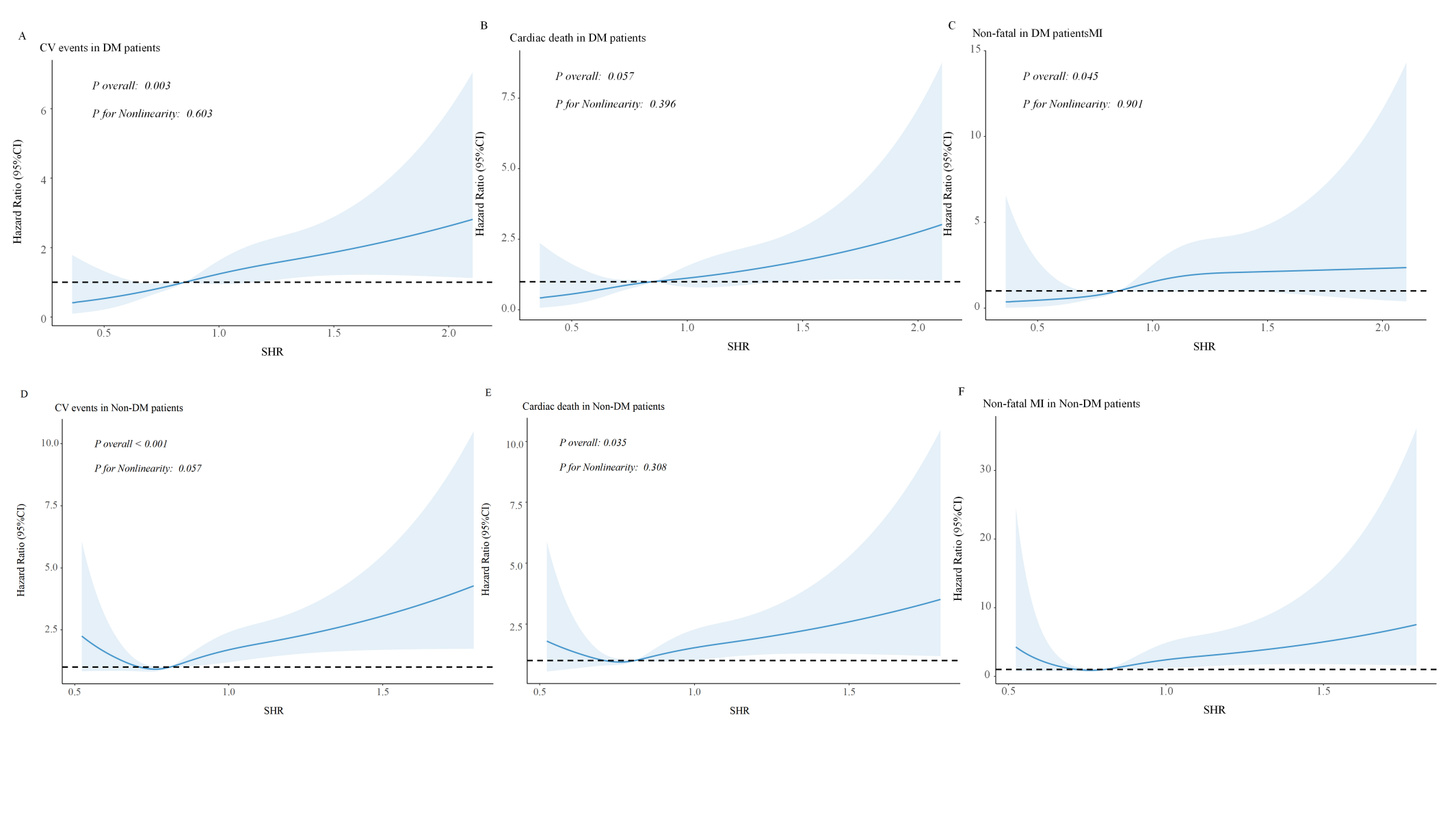
**RCS curves for (A), CV events (B), Cardiac death (C). Non-fatal MI in DM patients; (D), CV events (E), Cardiac death (F). Non-fatal MI in Non-DM patients; CV events cardiovascular events, SHR stress hyperglycemia ratio, DM diabetes mellitus
